# Supplementary material for: Experiences of adolescents and youth with HIV testing and linkage to care through the Red Carpet Program (RCP) in Kenya
Source: PLoS One. 2024 Jan 19;19(1):e0296786. doi: 10.1371/journal.pone.0296786 (PMC10798534; doi:10.1371/journal.pone.0296786)
Supplement: S1 Fig — (DOCX) [file pone.0296786.s001.docx]

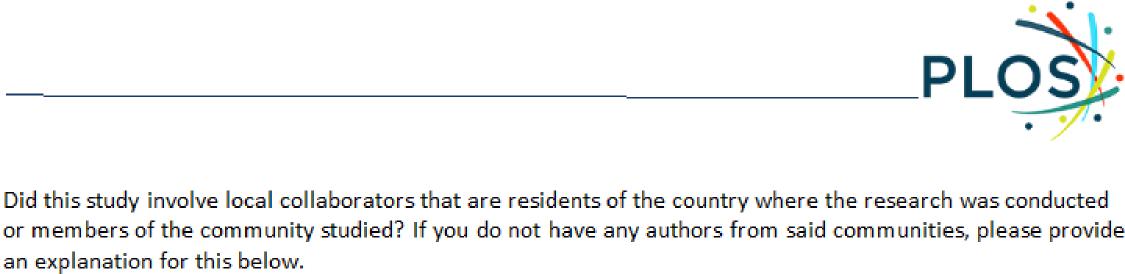
Inclusivity in global research

PLOS' policy on inclusivity in global research aims to improve transparency in the reporting of research performed outside of researchers' own country or community and ensures that PLOS publications reporting global research adhere to high standards for research ethics and authorship. Authors of relevant research articles may be asked to complete the questionnaire below, which outlines ethical, cultural, and scientific considerations specific to inclusivity in global research. This questionnaire may be requested when researchers have travelled to a different country to conduct research, if research uses samples collected in another country, research with Indigenous populations or their lands, or if research is on cultural artefacts. Researchers travelling to another country solely to use laboratory equipment will not normally be required to complete the questionnaire. However, the questionnaire can be requested at the journal's discretion for any submission — if you have been requested to complete this questionnaire by the PLOS journal you submitted to, please do so.

Please complete the questionnaire below and include this as a Supporting Information file with your manuscript. Note that if your paper is accepted for publication, this checklist will be published with your article in the supporting information files. Please ensure that you reference the checklist in the main body of your manuscript. We suggest adding a subsection 'Inclusivity in global research' to your Methods section and adding the following sentence: "Additional information regarding the ethical, cultural, and scientific considerations specific to inclusivity in global research is included in the Supporting Information (SX Checklist)"

The questions have been designed to be applicable to a wide range of study types, and there are subsections for both human subjects research and non-human subjects research. If any of the questions are not relevant to your research please mark them as "N/A" as appropriate.

Ethical considerations, permits and authorship

*This section is applicable to all research types.*

Provide details as to who granted permissions and/or consent for the study to take place in the Methods section of your manuscript. This should include the names of all ethics boards, governmental organizations, community leaders or other bodies that provided approval for the study. If individuals provided approval refer to these people by their role or title but do not list their name(s).

| Reported on page number six |
| --- |

If there were any deviations from the study protocol after approval was obtained please provide details of these changes in the Methods section of your manuscript.

There were no deviations from the protocol. Methods were implemented as proposed following ethical approval.


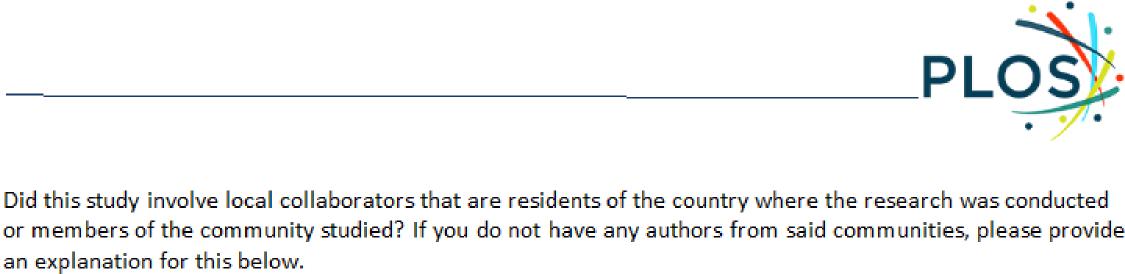


| The study team consisted of researchers from the local community and local EGPAF office. |
| --- |


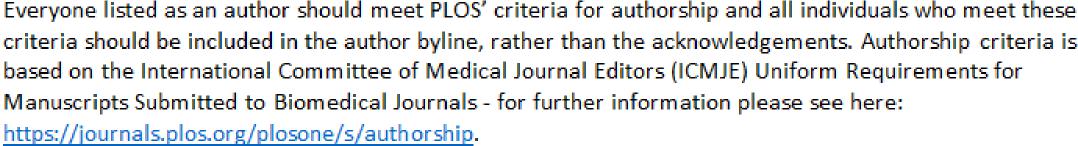


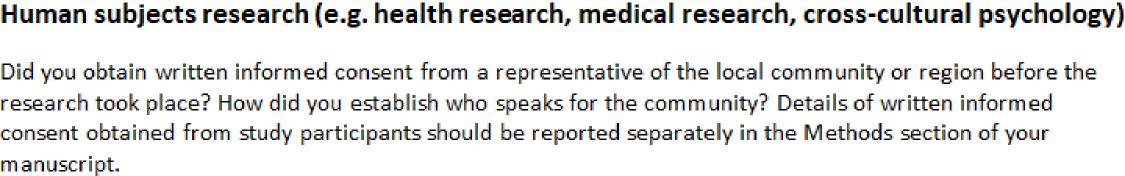


Written research consent was obtained from each participant ≥18 years of age prior to conducting FGDs. For participants aged 15-17 years, parental/guardian consent and AYLHIV assent were obtained. A waiver of parental/guardian consent was granted for mature minors 15-17 years old (married or pregnant/had children or being a head of households)


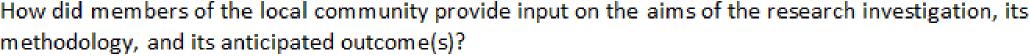


Members of the local community did not participate in the development of the protocol or implementation of the study. The findings were shared with the local facilities to inform their programming.


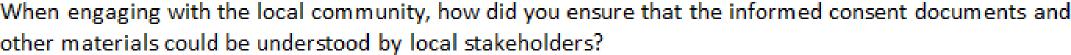


Research team received ethics and confidentiality training prior to any work on the study. The consent forms were developed at readability school grade 6 level and translated to the local language.


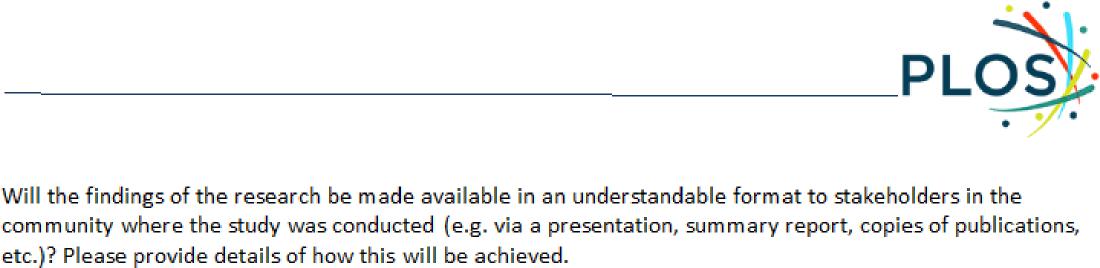


N/A

N/A

The study findings were shared with local facilities and local stakeholders as part of the implementation of the Red Carpet Program. The study findings have also informed technical assistance to youth focused programs in Kenya.


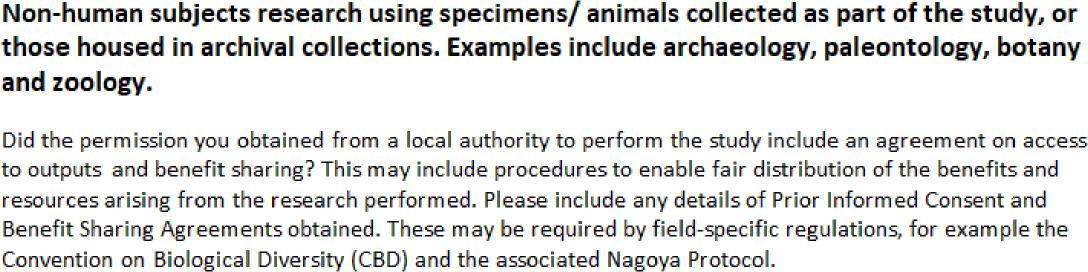


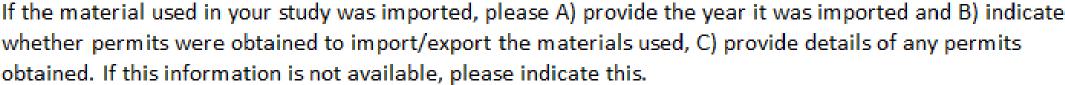


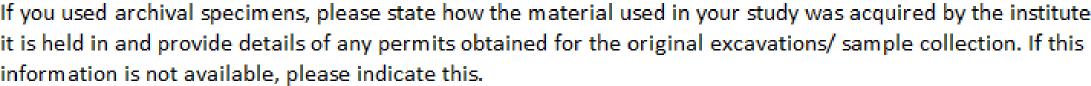


N/A


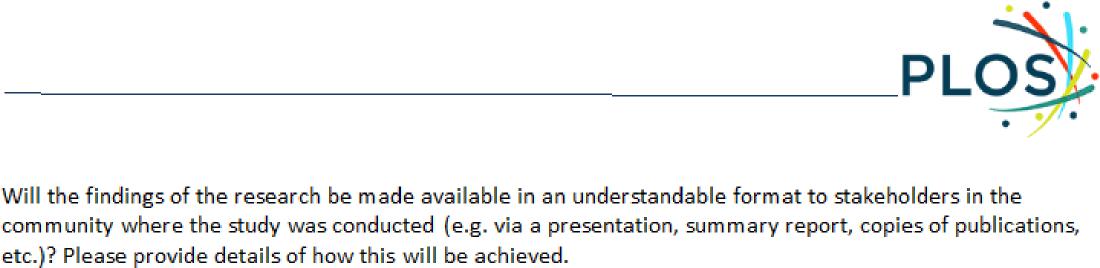


N/A

N/A

How was the potential cultural significance of the materials collected in your study to local communities considered in your research design? Were Indigenous peoples and/or local researchers and institutions involved with archaeological excavations / collection of specimens? If so, please provide a description of their involvement.

If your manuscript includes photographs of human remains please indicate whether authors obtained permission from descendants or affiliated cultural communities to do so.
