# Supplementary material for: Experiences of adolescents and youth with HIV testing and linkage to care through the Red Carpet Program (RCP) in Kenya
Source: PLoS One. 2024 Jan 19;19(1):e0296786. doi: 10.1371/journal.pone.0296786 (PMC10798534; doi:10.1371/journal.pone.0296786)
Supplement: S1 Appendix — (PDF) [file pone.0296786.s002.pdf]

S2 – Appendix

Research materials

A.

Experiences of Adolescents and Youth about HIV Testing and Linkage to Care in EGPAF Supported Health Facilities in Homa Bay County, Kenya.

Focus Group Discussion Guide.

Version 1.0

Date of the FGD __ __ / __ __ / __ __ __ __ (dd-mm-yyyy**)**
FGD Number _____
Site Number ____________________________________
Facilitator ____________________________________
Note- taker ____________________________________
Number of participants __ __ **Number of males __ Number of females __Start time** __ __ : __ __

Introduction:

☐ Following the greetings, introduce the Moderator and Note-taker.

☐ Explain that we are here to learn more about youth's perceptions of the linkage to HIV care services so that we can improve them. Briefly remind the participants of what the Red Carpet Program is. Confirm they have heard of it and/or understand what linkage to care is.

☐ Assign participant numbers on name tags to be referred to throughout the FGD. Explain that this helps protect their privacy and makes it easier for the note taker to capture what they say.

☐ Explain that we will begin with a short anonymous survey so we can find out who is participating in the discussion. Explain that no names or other identifying information should be written on the survey.

☐ Explain that the sessions will be audio recorded and once transcribed, the tapes will be destroyed. Explain that no names will be audio recorded. Explain that if anyone feels uncomfortable answering any of the questions, they can skip answering the question(s).

Discussion Guide:

For ***quantitative data***, try to record as many numbers as feasible so we can report the range; for ***qualitative data*** – try to summarize the answers with some direct anonymous quotes by participants, try record the gender for the person quoted. The answers can be verified with audio records.

HIV Testing

1. How long ago did you test positive for HIV? Can you tell me about your testing experience? (Probe to understand what made testing easy or challenging- location, access, staff, clinic setup, time to results, appointment scheduling, testing hours, peer coordinators, clinic support, etc.)
2. What encouraged you to get tested for HIV? (Probe to understand if testing was done because of provider recommendation, HIV education, prompts from family members, suggestions from friends, partner referral, sex without condom, etc.).
3. Did you ever get tested for HIV before this last positive test in your life?
4. In your opinion, what would encourage youth to test for HIV? (Probe to understand what could be done to encourage other youth to test)
5. It is known that more girls test for HIV than boys? Why do you think we test less boys and young men for HIV? How can we increase the number of young men tested for HIV?
6. Has anyone you ever encouraged someone else to be tested for HIV and is willing to share this experience?

HIV Support Services

1. As someone who received services from the Red Carpet program, how were your results and care handled on the day of your HIV diagnosis? (Probe to understand if staff were friendly, personable, did staff listen, offer advice, if the client felt supported)
2. How did you feel after receiving your diagnosis?
   a. Was a peer counselor (young person) available to discuss your diagnosis? Did you feel comfortable talking with him/her?
   b. What do you remember the most from the discussion about your HIV status?
   c. Was your safety and privacy maintained and how?
   d. How satisfied were you with your HIV counseling? (Probe with suggestions – satisfied, non-satisfied, somewhat satisfied and why)
   e. Do you feel that you have enough information about HIV? (Probe the satisfaction with the knowledge about HIV transmission, protection methods, knowledge about medication for HIV, knowledge about living with HIV in general, HIV care etc.)
3. At the time of your leaning about your HIV status, was there anything you wanted to talk about but couldn't? What was it?
   a. How easy was it for you to start your care and make your first appointments?
   b. Has your comfort level changed since your diagnosis to your first care appointment? If so, can you tell how it's different (Probe whether the change in personnel has affected the client)
   c. What and/or who makes you feel comfortable when you come in for your clinic visits?
   d. At your clinic visits now, is there anything you want to talk about but feel that you can't? What is it?
   e. How do you feel about the clinic staff, Red Carpet program coordinators, peer educators and other staff interacting with you during clinic visits, e.g. clinic exams, counseling, health education? (Probe to understand if they feel comfortable, safe, respected, supported, etc.)

f. Ask the youth to provide examples of how clinic staff interacted with them, place focus on youth friendly approach (e.g. being non-judgmental, welcoming, interested in young people's lives, etc.)
g. Do you have preferences to receive care from clinic staff of your own gender? If so, what part of your care?

4. How do you feel about the HIV services offered at this facility? (clinic exams, counseling, health education)
   a. What are some of the services that you really like at this facility?
   b. What are some of the services that you wish they would change? (Probe to understand how they could be improved)
   c. What are some other services you think youth would like to receive that do not exist here?
5. What can be done at the clinic or among clinic staff to improve your experiences?
6. What could the clinic facility staff do differently to improve how they provide HIV services for youth?

Disclosure and Disclosure Support

1. Do you know what it means to disclose your HIV status? Can you tell me more about what it means to you?
   a. Who do you feel most important disclosing your HIV status to?
2. Have you ever received any type of support to help you disclose your HIV status?
   a. Has anyone here disclosed their HIV status to another person and is willing to tell us about that experience?
   b. What type of response did have you had with disclosing your HIV status to another person? Who have you disclosed to? Family? Friend? Significant other? Teacher? Church leader?
   c. How many have told family/friends? How did it go?
   d. How was your disclosure experience overall? What would you change about it?
3. Who would you like to disclose your HIV status to that you haven't yet? How comfortable would you be disclosing your status to someone?
   a. What kind of support would help you to handle disclosure?
4. What kind of information and counseling, support and services do you get around partner relationships, protection (condoms), family planning, etc.?
5. What additional counseling, support and services would you like to receive?
6. Where in your community would you look to find support for disclosure outside of our health care facility? (Probe to see if school, church, family are options and what types of support is provided in each context.)
   a. What are the attitudes towards HIV among your peers and family?
   b. What are the attitudes towards HIV in your community?

Retention in Care and Referral Services

1. Has anyone ever missed an appointment at the clinic? (Probe to see if there are some who missed appointments regularly and some who missed them occasionally)

   a. What happened after you missed the appointment?
   b. Did someone from the clinic reach out to you? How long after the missed appointment?
   c. How do you remember your appointments? How would you like someone to remind you about coming to clinic?
2. Have you ever been referred to another services, providers or clinics – such as tuberculosis clinic, malnutrition clinic, social services, reproductive health services? How did the referral process work out for you?
3. Have you been referred to a peer support group? Did you join peer support group?
4. How can we help young people who learned about their diagnosis to stay in care? If someone is missing coming back to clinic, lost to follow up, or struggling to stay in care, how can the facility provide support?

Adherence

1. How long have you been on ARVs? When you started treatment, what information were you given about your medication? (For example, length of treatment, why treatment is important, and consequences of missing doses, etc.)
2. Has anyone here struggled with taking their HIV medications and is willing to share their experience?
3. What are some of the barriers that prevent you or others from adhering to your medicines? What are the reasons you might skip taking medications?
   a. How often do you forget? What kinds of experiences if any with side effects have you had?
   b. What helps you remember to take your medications?
   c. What would happen if you stop or skip frequently taking your HIV medicines? (Probe the knowledge about other infections, AIDS, HIV viral load, HIV resistance, CD4 cell counts and HIV transmission risks)
   d. What motivates you to take your medicines? (Probe the knowledge and attitudes about living positive, decreased risk of HIV transmission to a partner and a child)
   e. What support would you like to receive to help with adherence to your treatment?
4. Are there any challenges to getting your medications? (Probe individual, family related, school/life schedules, community)
5. In your opinion, can talking with other people living with HIV help being adherent?
6. Does talking with clinic staff during your appointments help with your adherence to medications when at home?
7. How do you feel about taking daily HIV medication for the rest of your life? Do you think it will be easy or difficult to do so? Why?
8. If the medicines were given as a shot once every two or three months – would you prefer it to taking daily pills?

End time __ __ : __ __

Annex B

Red Carpet Program Focus Group Discussion Anonymous Survey

THIS SURVEY IS CONFIDENTIAL
YOUR ANSWERS CANNOT AND WILL NOT BE LINKED TO YOU

Please DO NOT write your name on this questionnaire

This is a voluntary and anonymous confidential survey. We are not collecting any personal identifying information from you.

1. What is your gender?

   ☐Male ☐Female ☐Prefer not to state

2. How old are you? Age ranges (in years): _______

3. Are you currently in school? ☐ yes ☐ no

   If yes, how many years completed? _____

4. What is the highest level of school you completed?
   ☐ primary ☐ secondary ☐ college/university ☐ other……..specify

5. Do you have a job? ☐ yes ☐ no

6. How long have you known that you have HIV? ______ (number of months/years)

7. What is the name of the sub-county and facility where you receive your HIV care?
   ____

8. Are you currently taking ARV medications for HIV? ☐ yes ☐ no
